# Extracorporeal shockwave therapy shows a number of treatment related chondroprotective effect in osteoarthritis of the knee in rats

**DOI:** 10.1186/1471-2474-14-44

**Published:** 2013-01-28

**Authors:** Ching-Jen Wang, Shan-Ling Hsu, Lin-Hsiu Weng, Yi-Chih Sun, Feng-Sheng Wang

**Affiliations:** 1 Department of Orthopedic Surgery, Chang Gung University College of Medicine Kaohsiung Chang Gung Memorial Hospital, Kaohsiung, Taiwan; 2 Department of Medical Research, Chang Gung University College of Medicine Kaohsiung Chang Gung Memorial Hospital, Kaohsiung, Taiwan

**Keywords:** Shockwave, Number of treatment, Chondroprotective, Osteoarthritis, Knee, Rats

## Abstract

**Background:**

Extracorporeal shockwave therapy (ESWT) shows chondroprotective effect in osteoarthritis of the rat knees. However, the ideal number of ESWT is unknown. This study investigated the effects of different numbers of ESWT in osteoarthritis of the knee in rats.

**Methods:**

Forty-five male Sprague-Dawley rats were divided into five groups. Group I underwent sham arthrotomy without anterior cruciate ligament transection (ACLT) or medial meniscectomy (MM) and received no ESWT. Group II underwent ACLT + MM and received no ESWT. Group III underwent ACLT + MM, and received ESWT once a week for one treatment. Group IV underwent ACLT + MM and received ESWT twice a week for 2 treatments. Group V underwent ACLT + MM and received ESWT three times a week for 3 treatments. Each treatment consisted of 800 impulses of shockwave at 14 Kv to the medial tibia condyle. The evaluations included radiographs of the knee, histomorphological examination and immunohistochemical analysis at 12 weeks.

**Results:**

At 12 weeks, group II and V showed more radiographic arthritis than groups I, III and IV. On histomorphological examination, the Safranin O matrix staining in groups III and IV are significantly better than in groups II and V, and the Mankin scores in groups III and IV are less than groups II and V. Groups III and IV showed significant decreases of Mankin score and increase of Safranin O stain as compared to group I. Group V showed significant increases of Mankin score and a decrease of Safranin O stain as compared to group II. In articular cartilage, group II showed significant increase of MMP13 and decrease of collagen II as compared to group I. Groups III and IV showed significant decrease of MMP13 and increase of collagen II as compared to group I. Group V showed significant increase of MMP13 and decrease of collagen II as compared to group II. In subchondral bone, vWF, VEGF, BMP-2 and osteocalcin significantly decreased in groups II and V, but increased in groups III and IV relative to group I.

**Conclusions:**

ESWT shows a number of treatment related chondroproctective effect in osteoarthritis of the knee in rats.

## Background

Osteoarthritis (OA) of the knee has long being considered primarily a cartilage disease associated with cartilage loss and degradation. However, OA is usually accompanied by changes in the subchondral and periarticular bone such as sclerosis, bone cyst and osteophyte formation [1,2]. The relationship between the subchondral bone changes and the initiation and progression of OA is still debated [3-5]. Emerging evidence shows that bone turnover increases in patients with osteoarthritis with subchondral bone loss in the early stage of OA and bone formation with osteophytes in the late stage [6-9]. Some authors proposed the potential role of subchondral bone changes in the initiation and progression of OA [10,11]. It was suggested that increased subchondral bone stiffness reduces the ability of knee joint to dissipate the load and distribute the forces within the joint, and increases the force load on the overlying articular cartilage, which in turn accelerates the cartilage damage over time [6,9]. Therefore, the functional integrity of the articular cartilage depends on the mechanical properties of the subchondral bone. For early osteoarthritis of the knee, the initial focus of treatment on articular cartilage or in subchondral bone remains controversial.

Extracorporeal shockwave therapy (ESWT) has shown effectiveness in many orthopedic disorders including soft tissue tendinopathy and non-union of long bone fractures [12,13]. In addition, many studies reported positive effects of ESWT in various arthritic joints in animals [14-18]. Other studies demonstrated that ESWT is chondroprotective in the initiation of OA changes of the knee [19], and induces regression or retardation of established OA changes of the knee in rats [20]. The ESWT dosages in the studies were based on the results of a pilot study that demonstrated 800 impulses of shockwave at 14 Kv applied to the subchondral bone of the medial tibia condyle showed better effects than 200, 400 and 1200 impulses in small animals. However, the optimal dosage and the ideal number of ESWT in osteoarthritis of the knee are unknown. Furthermore, many studies reported a dose-related effect of ESWT in bone [21], tendon [22], epigastric skin flap [23], tenocyte [24], and cells [25]. We hypothesized that the effect of ESWT in osteoarthritis of the knee may be related to the number of ESWT treatment. The purpose of this study was to investigate the effect of different numbers of ESWT treatment in osteoarthritis of the knee in rats.

## Methods

The Institutional Review Board on animal experiment of Chang Gung Memorial Hospital, Taiwan approved this study. All studies were performed in accordance with the guidelines in the study and the care of animals in experiment.

### Study design

This study was performed in 45 male Sprague-Dawley rats of 10-week old with body weight ranging from 275 mg to 315 mg. The anterior cruciate ligament transected (ACLT) and medial meniscectomized (MM) osteoarthritis knee model in rat was used [8]. The animals were divided into five groups with 9 rats in each group. Group I was the control and underwent sham arthrotomy of the knee without ACLT or MM and received no ESWT. Group II underwent ACLT and MM but no ESWT. Group III underwent ACLT + MM and received ESWT once a week for one treatment. Group IV underwent ACLT and MM and received ESWT twice a week for two treatments. Group V underwent ACLT and MM and received ESWT three times a week for three treatments. Radiographs of the knee in anteroposterior and lateral projections were performed at 0 and 12 weeks. Radiographs of the knee were obtained to assess bony appearance, focal osteoporosis, narrowing of joint space and spur formation. The animals were sacrificed at 12 weeks and the knee specimens were subjected to histomorphological examination and immunohistochemical analysis.

### Anterior cruciate ligament transection and medial meniscectomy

The animals were sedated with intra-peritoneal phenobarbital injection (50 mg/Kg body weight). The left knee was prepared and draped in surgically sterile fashion. A straight anterior skin incision was made and the knee joint was opened through medial parapatellar arthrotomy. The anterior cruciate ligament was transected with a scalpel. Medial meniscectomy was performed by excising the entire medial meniscus. The knee was irrigated and the wound was closed in routine fashion. The animals were returned to the housing cages and were under the care of the veterinarian.

### Shockwave application

**Figure 1:**
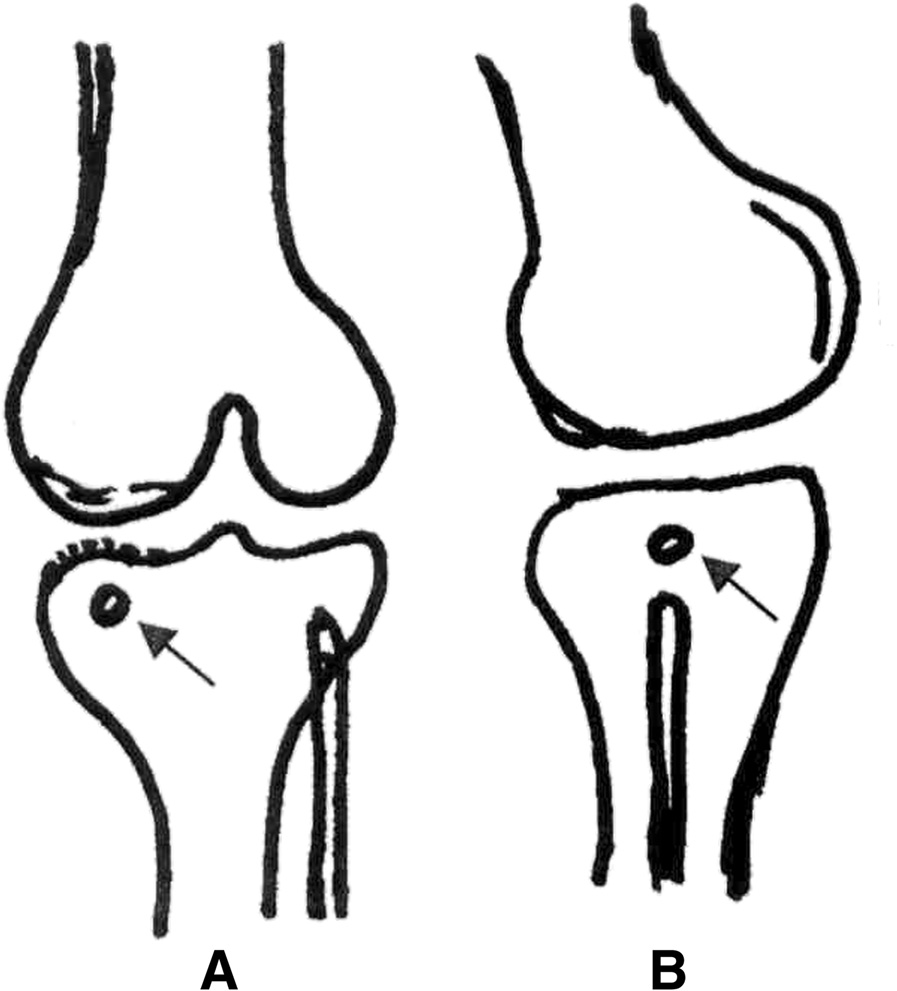
A knee sketch shows the location of shockwave application in A-P (1-A) and lateral view (1-B).

ESWT was administered in groups III, IV and V in one week after knee surgery when the wound healed. The animals were sedated with intra-peritoneal phenobarbital injection (50 mg/Kg body weight). The source of shockwave is from an OssaTron orthotriptor (Sanuwave, Alpharetta, GA). The focus of shockwave treatment was the subchondral bone of the medial tibia condyle that was approximately 0.5 cm below the medial tibia plateau in anteroposterior view and 0.5 cm from the medial skin in lateral view (Figure 1). The depth of treatment was confirmed with the laser indicator of the shockwave device. Ultrasound gel was applied to the skin in contact with the shockwave tube. Each treatment consisted of the application of 800 impulses of shockwave at 14 Kv (equivalent to 0.22 mJ/mm^2^). ESWT was performed once in group III, twice in group IV and three times in group V. After ESWT, the animals were returned to the housing cages and are under the care of the veterinarian.

### Histomorphological examination

The animals were sacrificed at 12 weeks. The knee specimens including the articular cartilage and the subchondral bone of proximal tibia and distal femur were harvested. The specimens were decalcified and fixed in paraffin, and cut into 5-um thick sections using microderme and stained with heamtoxylin-eosin, Safranin-O, thionine and Alcian blue stains. The microscopic features of the articular cartilage included fissuring of the cartilage, chondrocyte proliferation, chondrocyte activity and chondrocyte apoptosis. The cartilage degradation was assessed by Mankin score that included cartilage structure, cartilage cells and tidemark integrity [26]. The matrix content was measured with Safranin O staining. The subchondral bone remodeling was evaluated with tissue distributions including cortical bone, cancellous bone and fibrous tissue.

### Immunohistochemical analysis

The specimens were fixed in 4% PBS-buffered paraformaldehyde for 48 hours and decalcified in PBS-buffered 10% EDTA. Decalcified tissues were embedded in paraffin. The specimens were cut longitudinally into 5-um thick sections and transferred to poly-lysine-coated slides. Sections of the specimens were immunostained with specific reagents for vWF (von Willebrand Factor), VEGF (vessel endothelial growth factor), BMP-2 (bone morphogenic protein 2) and osteocalcin in subchondral bone, and MMP13 (matrix metalloproteinase 13) and collagen II in articular cartilage (Santa Cruz Biotechnology Inc, CA, USA). The immuno-reactivity in specimens was demonstrated using a horseradish peroxidase (HRP)-3′-, 3′-diaminobenzidine (DAB) cell and tissue staining kit (R & D Systems, Inc. Minneapolis, MN, USA). The immuno-activities were quantified from five areas in three sections of the same specimen using a Zeiss Axioskop 2 plus microscope (Carl Zeiss, Gottingen, Germany). All the images of each specimen were captured using a Cool CCD camera (SNAP-Pro c.f. Digital kit; Media Cybernetics, Sliver Spring, MD, USA). Images were analyzed using an Image-Pro® Plus image-analysis software (Media Cybernetics, Sliver Spring, MD, USA). The percentage of positive immuno-labeled cells over the total cells in each area was counted. A pathologist blinded to the nature of the study performed the measurements on all sections. The cartilage degradation was assessed with the measurements of MMP-13 and collagen II in the articular cartilage, and the subchondral bone remodeling by the measurements of vWF, VEGF, BMP-2 and osteocalcin.

### Statistical analysis

The results of this study were expressed in median with ranges. Group I data were used as the baseline for statistical comparison with other groups. The P-values were obtained using ANOVA and post hoc test with Bonferoni correction among five groups, and Mann-Whitney “*U*” test between two groups. A statistical significance was set at p < 0.05.

## Results

**Figure 2:**
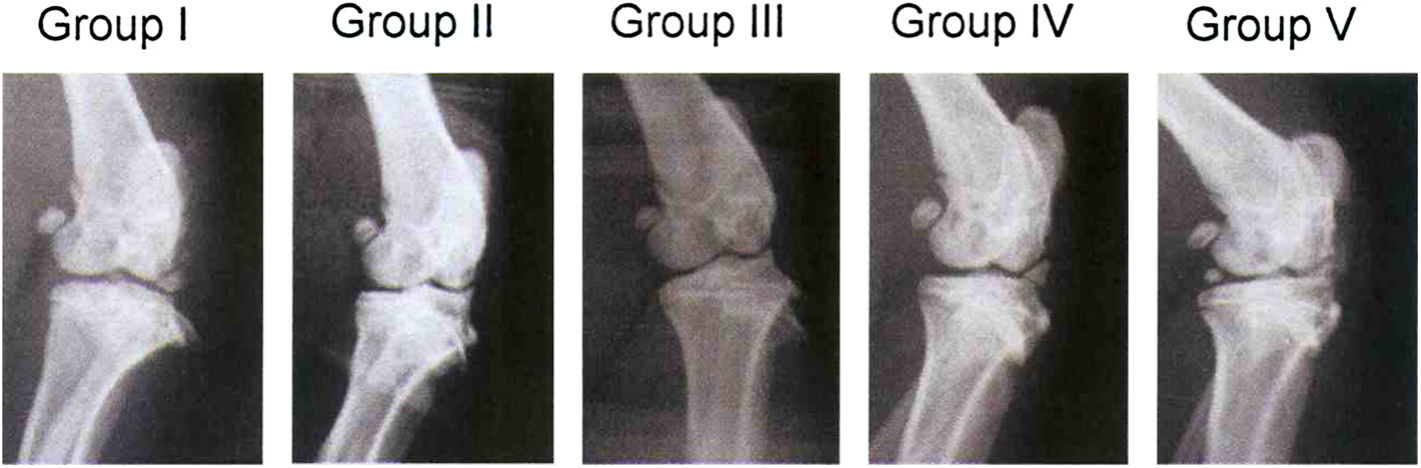
Radiographs of the knee at 12 weeks show more advanced osteoarthritis of the knee in groups II and V, and less arthritic changes in groups I, III and IV.

Radiographs of the knee showed no discernable difference among the 5 groups at 0 week. However, at 12 weeks, groups II and V showed more radiographic arthritis than groups I, III and IV (Figure 2).

**Figure 3:**
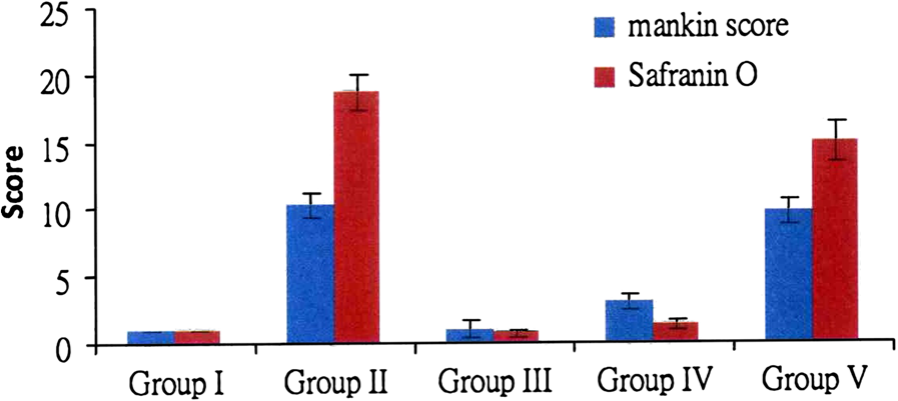
The results of Mankin score and Safranin O stain.

**Figure 4:**
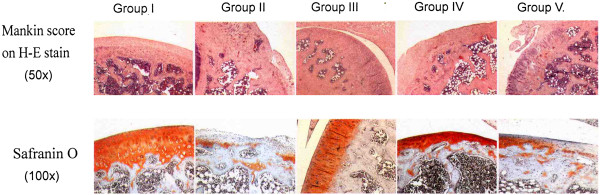
Microscopic features of the articular cartilage and subchondral bone in histomorphological examination.

The results of Mankin score and Safranin O matrix staining are summarized in Table 1 and Figure 3. The microscopic features are shown in Figure 4. The Safranin O matrix staining in group III and IV is significantly better than in groups II and V, and the Mankin scores in groups III and VI are less than groups II and V. The figures reflect a decreased Safranin O staining and an increased Mankin score associated with progression of osteoarthritis of the knee. The knees that were treated with one and two ESWT treatments showed significantly better Mankin score and Safranin O staining than knees treated with three ESWT treatments.

**Table 1:** The results of Mankin score and Safranin O stain

**Mankin score**				
**Group I**	**Group II**	**Group III**	**Group IV**	**Group V**
1 (1-1)	10 (9-12)	1 (0-2)	3 (2-4)	10 (8-11)
	P1 = 0.0088	P2 = 1	P3 = 0.0742	P4 = 0.0102
		P5 = 0.0017	P6 = 0.0038	P7 = 0.6213
			P8 = 0.0705	P9 = 0.0022
				P10 = 0.0052
**Safranin O**				
**Group I**	**Group II**	**Group III**	**Group IV**	**Group V**
1 (1-1)	18.7 (16-20)	0.7 (0-1)	1.3 (1-2)	15 (12-17)
	P1 = 0.0056	P2 = 0.4226	P3 = 0.4226	P4 = 0.0117
		P5 = 0.0036	P6 = 0.0039	P7 = 0.1461
			P8 = 0.2302	P9 = 0.0087
				P10 = 0.0096

Group I: the control; Group II: ACLT + MM; Group III: ACLT + MM + ESWT 1×/week; Group IV: ACLT + MM + ESWT 2×/week; Group V: ACLT + MM + ESWT 3×/week.

ACLT: anterior cruciate ligament transection; MM: medial meniscectomy; ESWT: extracorporeal corporeal shockwave therapy.

P1: Group I vs Group II P2: Group I vs Group III P3: Group I vs Group IV.

P4: Group I vs Group V P5: Group II vs Group III P6: Group II vs Group IV.

P7: Group II vs Group V P8: Group III vs Group IV P9: Group III vs Group V.

P10: Group IV vs Group V.

The P values were obtained using ANOVA and post hoc test with Bonferoni correction among five groups, and the Mann-Whitney *U* test between two groups.

**Figure 5:**
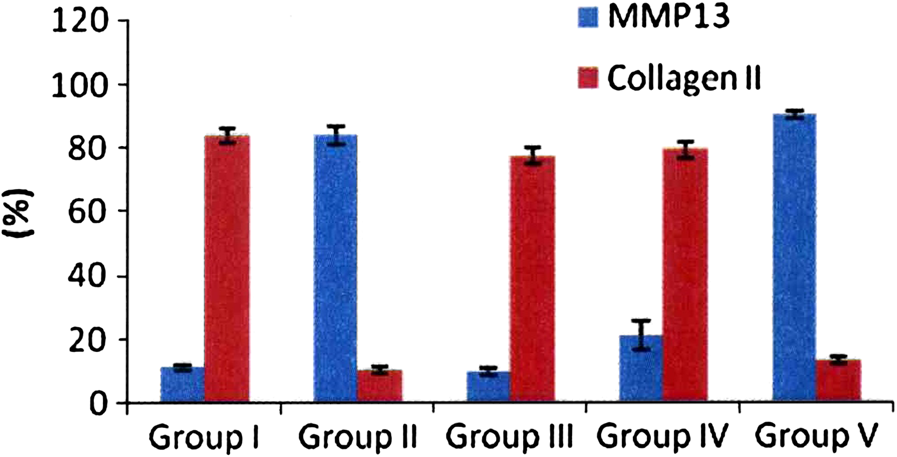
The results of MMP13 and collagen II in articular cartilage.

**Figure 6:**
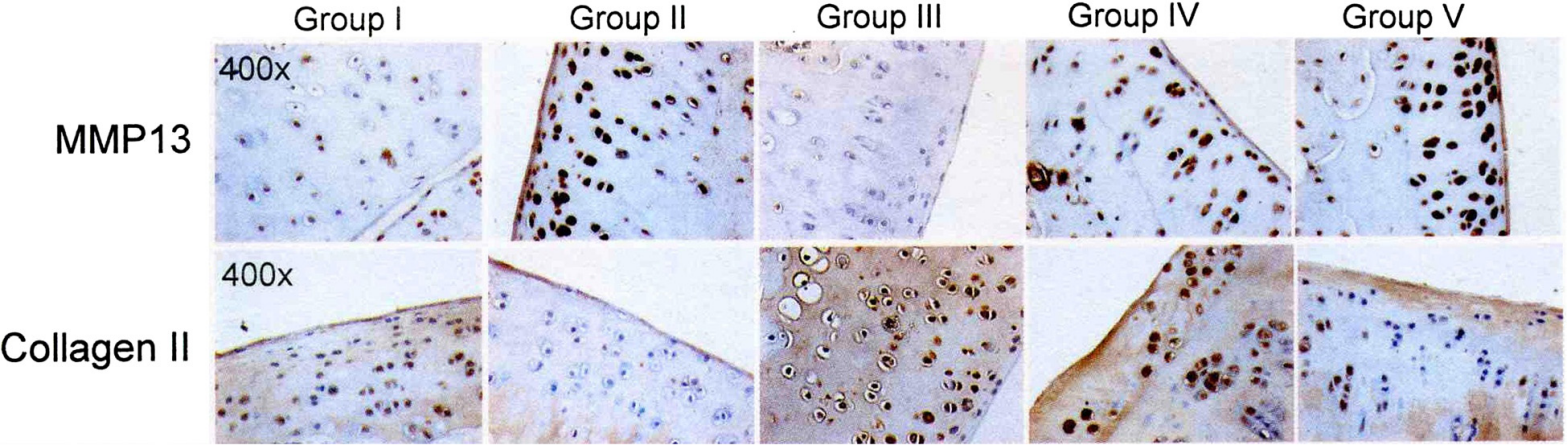
Microscopic features of MMP13 and collagen II in articular cartilage in immunohistochemical stains.

The results of MMP13 and collagen II of the articular cartilage are summarized in Table 2 and Figure 5. The microscopic features are shown in Figure 6. Group II showed significant increase of MMP13 and decreases of collagen II as compared to group I indicating the progression of the knee arthritis. The changes of MMP-13 and collagen II in groups III and IV were comparable to group I, however, the changes in group V are closely similar to group II.

**Table 2:** The results of MMP13 and collagen II in articular cartilage

**MMP13 (%)**				
**Group I**	**Group II**	**Group III**	**Group IV**	**Group V**
11.2 (9.4-12)	83.1 (79.5-89.3)	9.4 (7.8-11.3)	22.9 (11.8-27.3)	89.1 (88.5-92.4)
	P1 = 0.0008	P2 = 0.3467	P3 = 0.1643	P4 < 0.0001
		P5 = 0.0005	P6 = 0.0008	P7 = 0.1578
			P8 = 0.1307	P9 < 0.0001
				P10 = 0.0027
**Collagen II (%)**				
**Group I**	**Group II**	**Group III**	**Group IV**	**Group V**
83.7 (80.4-87.5)	10.4 (7.6-11.7)	76 (73.7-82.1)	77.5 (76-83.7)	12.4 (11.5-14.5)
	P1 < 0.0001	P2 = 0.1142	P3 = 0.2007	P4 = 0.0001
		P5 = 0.0002	P6 = 0.0001	P7 = 0.1319
			P8 = 0.6280	P9 = 0.0005
				P10 = 0.0003

P1: Group I vs Group II P2: Group I vs Group III P3: Group I vs Group IV.

P4: Group I vs Group V P5: Group II vs Group III P6: Group II vs Group IV.

P7: Group II vs Group V P8: Group III vs Group IV P9: Group III vs Group V.

P10: Group IV vs Group V.

The P values were obtained using ANOVA and post hoc test with Bonferoni correction among five groups, and the Mann-Whitney *U* test between two groups.

**Figure 7:**
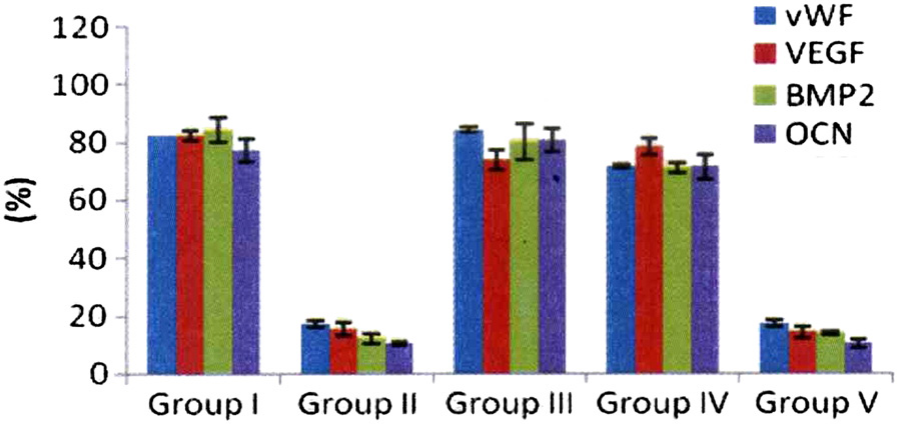
The results of vWF, VEGF, BMP-2 and osteocalcin in subchondral bone.

**Figure 8:**
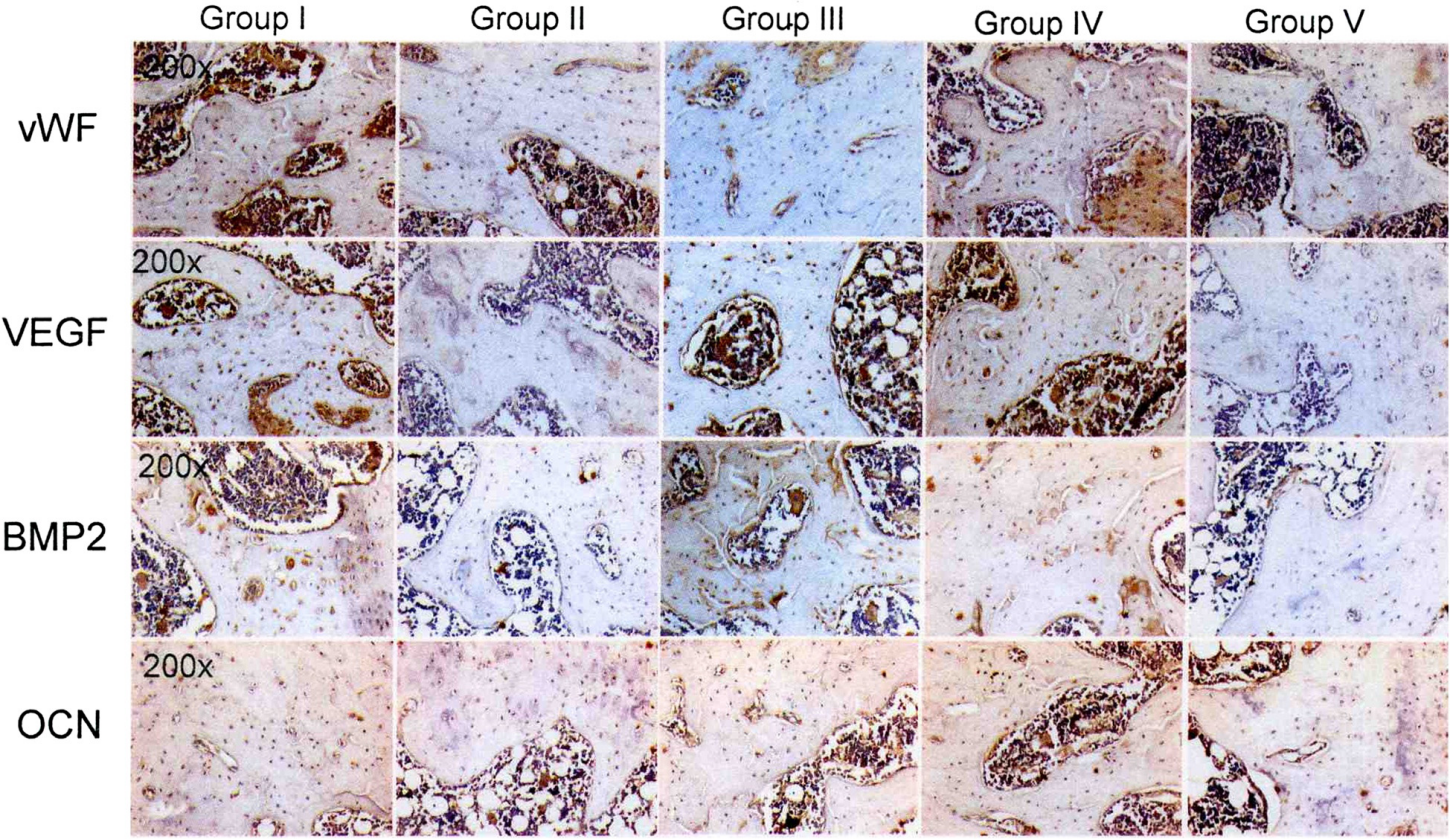
**Microscopic features of vWF, VEGF.** BMP-2 and osteocalcin in subchondral bone in immunohistochemical stains.

The results of vWF, VEGF, BMP-2 and osteocalcin in subchondral bone are summarized in Table 3 and Figure 7. The microscopic features are shown in Figure 8. Group II showed significant decreases of vWF, VEGF, BMP-2 and osteocalcin as compared to group I. Group III and IV showed significant increases of vWF, VEGF, BMP-2 and osteocalcin with the data comparable to group I. However, group V showed significant decreases of vWF, VEGF, BMP-2 and osteocalcin comparable to group II. It appears that one or two ESWT treatments showed beneficial effects, however, three ESWT treatments caused deteriorating changes.

**Table 3:** The results of vWF, VEGF, BMP-2 and osteocalcin in subchondral bone

**vWF (%)**				
**Group I**	**Group II**	**Group III**	**Group IV**	**Group V**
81.9 (78.9-86.8)	16.2 (15.4-19.5)	84.8 (79.7-89.1)	73.8 (62.9-78.3)	17.6 (13.1-20.3)
	P1 = 0.0001	P2 = 0.8696	P3 = 0.1253	P4 < 0.0001
		P5 = 0.0012	P6 = 0.0044	P7 = 0.9899
			P8 = 0.1156	P9 = 0.0004
				P10 = 0.0022
**VEGF (%)**				
**Group I**	**Group II**	**Group III**	**Group IV**	**Group V**
82.8 (79-84.6)	15.4 (11.2-19.5)	72.5 (68.6-80.4)	78.3 (73.8-83.5)	13.1 (11.3-17.6)
	P1 = 0.0001	P2 = 0.1239	P3 = 0.3432	P4 < 0.0001
		P5 = 0.0003	P6 = 0.0001	P7 = 0.6777
			P8 = 0.3540	P9 = 0.0005
				P10 = 0.0001
**BMP2 (%)**				
**Group I**	**Group II**	**Group III**	**Group IV**	**Group V**
84.4 (77.1-91)	11.2 (9.4-15.4)	74.6 (73.1-89.1)	72.9 (68.3-73.8)	13.1 (12.4-14.6)
	P1 = 0.0008	P2 = 0.4670	P3 = 0.0729	P4 = 0.0026
		P5 = 0.0027	P6 < 0.0001	P7 = 0.5312
			P8 = 0.2893	P9 = 0.0054
				P10 = 0.0002
**OCN (%)**				
**Group I**	**Group II**	**Group III**	**Group IV**	**Group V**
79.6 (69.6-82.2)	10.5 (9.4-11.2)	76.7 (76.1-87.8)	73.8 (62.9-77.3)	9.1 (8.5-12.4)
	P1 = 0.0029	P2 = 0.6008	P3 = 0.3741	P4 = 0.0016
		P5 = 0.0025	P6 = 0.0045	P7 = 0.8010
			P8 = 0.2002	P9 = 0.0014
				P10 = 0.0029

P1: Group I vs Group II P2: Group I vs Group III P3: Group I vs Group IV.

P4: Group I vs Group V P5: Group II vs Group III P6: Group II vs Group IV.

P7: Group II vs Group V P8: Group III vs Group IV P9: Group III vs Group V.

P10: Group IV vs Group V.

The P values were obtained using ANOVA and post hoc test with Bonferoni correction among five groups, and the Mann-Whitney *U* test between two groups.

## Discussion

The results of the current study showed that ESWT protects the articular cartilage degradation and improves subchondral bone remodeling in the initiation of osteoarthritis of the knee in rats. The results are in agreement with prior studies that shockwave has chondroprotective effect in OA knee in rats [19,20], The subchondral bone remodeling relies on the balance between bone formation by osteoblast and bone resorption by osteoclast, and is influenced by the subchondral bone mass and bone strength. The effects on articular cartilage were supported by the changes of Mankin score, Safranin O stain, MMP-13 and collagen II. MMP-13 is involved in cartilage turnover and cartilage pathophysiology associated with osteoarthritis. Collagen II is the base of articular cartilage and hyaline cartilage that form fibrillar network of collagen that allows cartilage to entrap the proteoglycan aggregate and provide tensile strength to the tissue. The effects of ESWT in subchondral bone remodeling were supported by the increased vascularization manifested by the changes of vWF, eNOS and VEGF, and osteogenesis manifested by the changes in BMP-2, alkaline phosphatase and osteocalcin. vWF is a large glycoprotein in plasma and endothelium and a positive vWF is indicative of new vessel formation. eNOS is an immune transmitter and vasodilator in tissue healing . VEGF is a protein that stimulates vasculogenesis and angiogenesis and an indication of increased vascular permeability and microvascular activities including the angiogenic growth of new vessels. BMP-2 is osteoinductive and induces osteoblast differentiation. Alkaline phosphatase is a hydrolase enzyme responsible for dephosphorylation. Osteocalcin is pre-osteoblastic and bone-building in nature.

The current study also demonstrated that ESWT has a number of treatment related effect in osteoarthritis of the knee. Many studies reported dose-dependent effects of ESWT in different tissues [21-25]. ESWT showed a dose-related enhancement in bone mass and bone strength after fracture of the femur in rabbits [21]. Other study reported a dose-related effect of shockwaves on rabbit tendon Achilles [22]. In a murine skin flap model, ESWT between 500 and 2500 impulses showed a dose-dependent effect in epigastric flap survival [23]. ESWT also showed dose-dependent changes in cell viability, MMP 1, 2 and 3 and IL-6 on cultured tenocytes [24]. Another study stated that one of the most important aspects to be considered is not the total number of impulses used but the energy level of the shockwaves, thus confirming that ESWT has a dose-dependent effect on osteoblast cells [25]. An optimal ESWT dosage may result in beneficial effects, whereas, overdose of ESWT can cause deteriorating effect and damage to the subchondral bone and articular cartilage of the knee. The current study demonstrated that one or two ESWT treatments showed beneficial effect, however, three ESWT treatments caused deteriorating results. Additional studies including clinical trial are needed to validate the ideal numbers of ESWT for osteoarthritis of the knee in humans.

The exact mechanism of ESWT remains unknown. Recent studies showed that ESWT induces the ingrowth of neovascularization and up-regulation of angiogenic and osteogenic growth factors that leads to improvement in blood perfusion and tissue regeneration [27]. The innovative findings in this study may unveil a new concept in the management of osteoarthritis of the knee by shifting the initial treatment focus from the articular cartilage to the subchondral bone. Furthermore, ESWT has the potential used in the treatment of osteoarthritis of the knee.

There are limitations in this study. The data obtained from this study were based on experiments in small animals. The results may differ in larger animals or human subjects. The dose conversion (energy level and the total energy) from small animals to larger animals or human subjects must be validated with additional studies including clinical trial. This study demonstrated that the effect of ESWT is related to the number of treatment, but not necessarily the dosage in knee OA, and the optimal ESWT dosage and the ideal numbers of ESWT treatment remains unknown. Furthermore, different manufacture companies used different indices of shockwave parameters, and the dose conversion formula among the different devices are not readily available at the present time.

## Conclusion

ESWT shows a number of treatment related chondroprotective effects and improves subchondral bone remodeling in the initiation of osteoarthritis of the knee in rats. Additional studies are needed to validate the optimal ESWT dosage and the number of treatment for osteoarthritis of the knee.

## Competing interests

The authors declared that they did not receive any honoraria or consultancy fees in writing this manuscript. No benefits in any form have been received or will be received from a commercial party related directly or indirectly to the subject of this article. One author (CJW) has served as a member of scientific advisory committee of Sanuwave, Alpharetta, GA that is irrelevant to the current study. The remaining authors declare no conflict of interest.

## Authors’ contributions

C-JW participated in the study with primary responsibility in conception and design drafting, overview the entire study, and data collection and analysis, literature review, reference search, draft writing and critically revised the manuscript and read proof of the final manuscript. S-LH participated in the study with primary responsibility in the supervision and performance of animal experiments, reference search and read proof the final manuscript. L-HW participated in this study with the primary responsibility to supervise animal experiment, reference search, reference review and read proof the final manuscript. Y-CS participated in the study with primary responsibility in performing animal experiment including application of shockwave and laboratory studies including histomorphological examination and immunohistochemical analysis, and. read proof the final manuscript. F-SW participated in this study with the primary responsibility to supervise histomorphological examination and immunohistochemical analysis, reference search and read proof the final manuscript. All authors read and approved the final manuscript.

## Pre-publication history

The pre-publication history for this paper can be accessed here:

http://www.biomedcentral.com/1471-2474/14/44/prepub

## References

[B1] Lane NE Nevitt MC Osteoarthritis, bone mass, and fractures: how are they related? Arthritis Rheum 2002 46 1 4 10.1002/1529-0131(200201)46:1<1::AID-ART10068>3.0.CO;2-P 11817580

[B2] Oettmeier R Abendroth K Osteoarthritis and bone: osteologic types of osteoarthritis of the hip Skeletal Radiol 1989 18 165 174 10.1007/BF00360962 2749285

[B3] Burr DM Schaffler MB The involvement of subchondral mineralized tissues in osteoarthrosis: quantitative microscopic evidence Micros Res Tech 1997 37 343 357 10.1002/(SICI)1097-0029(19970515)37:4<343::AID-JEMT9>3.0.CO;2-L 9185156

[B4] Burr DB The importance of subchondral bone in osteoarthrosis Curr Opin Rheumatol 1998 10 256 262 10.1097/00002281-199805000-00017 9608330

[B5] Radin EL Rose RM Role of subchondral bone in the initiation and progression of cartilage damage Clin Orthop 1986 213 34 40 3780104

[B6] Dedrick DK Goulet R Huston L Goldstein SA Bole GG Early bone changes in experimental osteoarthritis using microscopic computed tomography J Rheumatol 1991 27 Suppl 44 45 2027128

[B7] Hayami T Funaki H Yaoeda K Mitui K Yamagiwa H Tokunaga K Hatano H Kondo J Hiraki Y Yamamoto T Duong le T Endo N Expression of the cartilage derived anti-angiogenic factor chondromodulin-I decreases in the early stage of experimental osteoarthritis J Rheumatol 2003 30 2207 2217 14528519

[B8] Hayami T Pickarski M Zhuo Y Wesolowski GA Rodan GA le Duong T Characterization of articular cartilage and subchondral bone changes in the rat anterior cruciate ligament transection and meniscectomized models of osteoarthritis Bone 2006 38 2 234 243 10.1016/j.bone.2005.08.007 16185945

[B9] Ratcliffe A Seibel MJ Biochemical markers of osteoarthritis Curr Opin Rheumatol 1990 277 21352 21360 10.1097/00002281-199002050-00014 2265073

[B10] Muraoka T Hagino H Okano T Enokida M Teshima R Role of subchondral bone in osteoarthritis development: a comparative study of two strains of guinea pigs with and without spontaneously occurring osteoarthritis Arthritis Rheum 2007 56 10 3366 3374 10.1002/art.22921 17907190

[B11] Dieppe P Subchondral bone should be the main target for the treatment of pain and disease progression in osteoarthritis Osteoarthritis Cartilage 1999 7 3 325 326 10.1053/joca.1998.0182 10329316

[B12] Wang CJ An overview of shock wave therapy in musculoskeletal disorders Chang Gung Med J 2003 26 220 232 12846521

[B13] Wang CJ Extracorporeal shockwave therapy in musculoskeletal disorders [Review] Journal of Orthopaedic Surgery & Research 2012 7 11 10.1186/1749-799X-7-11 22433113 PMC3342893

[B14] Dahlberg J Fitch G Evans RB McClure SR Conzemius M The evaluation of extracorporeal shockwave therapy in naturally occurring osteoarthritis of the stifle joint in dogs Vet Comp Orthop Traumatol 2005 18 3 147 152 16594445

[B15] Frisbie DD Kawcak CE Mcllwraith CW Evaluation of the effect of extracorporeal shock wave treatment on experimentally induced osteoarthritis in middle carpal joints of horses Am J Veterinary Res 2009 70 4 449 454 10.2460/ajvr.70.4.449 19335099

[B16] Mueller M Bockstahler B Skalicky M Mlacnik E Lorinson D Effects of radial shockwave therapy on the limb function of dogs with hip osteoarthritis Vet Rec 2007 160 22 762 765 10.1136/vr.160.22.762 17545646

[B17] Ochiai N Ohtori S Sasho T Nakagawa K Takahashi K Takahashi N Murata R Takahashi K Moriya H Wada Y Saisu T Extracorporeal shock wave therapy improves motor dysfunction and pain originating from knee osteoarthritis in rats Arthritis & Cartilage 2007 15 9 1093 1096 17466542 10.1016/j.joca.2007.03.011

[B18] Revenaugh MS Extracorporeal shock wave therapy for treatment of osteoarthritis in the horse: clinical application Vet Clin North Am Equine Pract 2005 21 3 609 625 10.1016/j.cveq.2005.09.001 16297724

[B19] Wang CJ Weng LH Ko JY Sun YC Yang YJ Wang FS Extracorporeal shockwave therapy shows chondroprotective effects in osteoarthritic Rat knee Arch Orthop Trauma Surg 2011 131 1153 1158 10.1007/s00402-011-1289-2 21387139

[B20] Wang CJ Weng LH Ko JY Wang JW Chen JM Sun YC Yang YJ Extracorporeal shockwave shows regression of osteoarthritis of the knee in rats J Surg Res 2011 171 601 608 10.1016/j.jss.2010.06.042 20851422

[B21] Wang CJ Yang KD Wang FS Chen HS Chen HH Hsu CC Shock wave therapy enhances bone mass and bone strength after fracture of the femur. A study in rabbits Bone 2004 34 225 230 10.1016/j.bone.2003.08.005 14751581

[B22] Rompe JD Kirpatrick CJ Kullmer K Schneitalle M Krischeck O Dose-related effects of shockwaves on rabbit tendon Achilles J Bone Joint Surg Br 1998 80 546 552 10.1302/0301-620X.80B3.8434 9619954

[B23] Kamelger F Oehlbauer M Piza-Katzer H Meirer R Extracorporeal shock wave treatment in ischemic tissues: what is the appropriate number of shock wave impulses? J Reconstructive Microsurgery 2010 26 2 117 121 10.1055/s-0029-1243296 20013593

[B24] Han SH Lee JW Guyton GP Parks BG Courneya JP Schon LC Effect of extracorporeal shock wave therapy on cultured tenocytes Foot Ankle Int 2009 30 2 93 98 10.3113/FAI.2009.0093 19254500

[B25] Martini L Fini M Giavaresi G Torricelli P DePretto M Rimondini L Giardino R Primary osteoblasts response to shock wave therapy using different parameters Artif Cells Blood Substit Immobil Biotechnol 2003 31 4 449 466 10.1081/BIO-120025415 14672419

[B26] Mankin HJ Dorfman H Lippiello L Zarins A Biochemical and metabolic abnormalities in articular cartilage from osteo-arthritic human hips: II. Correlation of morphology with biochemical and metabolic data J Bone Joint Surg Am 1971 53 523 537 5580011

[B27] Wang CJ Wang FS Yang KD Huang CS Hsu CC Shock wave therapy induces neovascularization at the tendon-bone junction. A study in rabbits J Orthop Res 2003 21 984 989 10.1016/S0736-0266(03)00104-9 14554209

